# Is parvovirus B19 infection upsurge in 2023–2024 associated with adverse pregnancy outcome?

**DOI:** 10.1002/uog.29301

**Published:** 2025-07-30

**Authors:** S. Prasad, A. Khalil, Y. Yinon, N. Regev, R. Brawura‐Biskupski‐Samaha, M. Massoud, N. Mazanowska, F. G. Sileo, F. Prefumo, P. Kosinski, J. Morales Rosello, F. D'Antonio, Collaborators, C. Ozan Ulusoy, D. Mohammed, M. Arora, A. Hegde, E. Klapholz, C. Berkovitz, K. Kosińska‐Kaczyńska, I. Rzucidło‐Szymańska, N. Szymecka‐Samaha, S. Ceffa

**Affiliations:** ^1^ Fetal Medicine Unit St George's University Hospitals NHS Foundation Trust, University of London London UK; ^2^ Vascular Biology Research Centre, Molecular and Clinical Sciences Research Institute St George's University of London London UK; ^3^ Fetal Medicine Unit, Liverpool Women's Hospital Liverpool UK; ^4^ Department of Obstetrics and Gynecology, Sheba Medical Center, Faculty of Medical and Health Sciences Tel Aviv University Tel Aviv Israel; ^5^ Department of Obstetrics, Perinatology and Neonatology Centre of Postgraduate Medical Education Warsaw Poland; ^6^ Department of Obstetrics and Gynaecology University Hospitals Lyon Lyon France; ^7^ Department of Obstetrics and Gynecology Institute of Mother and Child Warsaw Poland; ^8^ Prenatal Medicine Unit, Obstetrics and Gynaecology Unit, Department of Medical and Surgical Sciences for Mother, Child and Adult University of Modena and Reggio Emilia Modena Italy; ^9^ Obstetrics and Gynecology Unit IRCCS Istituto Giannina Gaslini Genova Italy; ^10^ Department of Obstetrics, Perinatology, Gynecology and Reproductive Medicine Medical University of Warsaw Warsaw Poland; ^11^ Department of Obstetrics and Gynecology La Fe University and Polytechnic Hospital Valencia Spain; ^12^ Center for Fetal Care and High‐Risk Pregnancy, Department of Obstetrics and Gynecology University “G. d'Annunzio” of Chieti‐Pescara Chieti Italy

**Keywords:** 2023–2024 outbreak, adverse pregnancy outcomes, fetal anemia, fifth disease, hydrops fetalis, intrauterine transfusion, maternal infection, parvovirus B19, perinatal mortality, pregnancy outcomes, slapped‐cheek syndrome

## Abstract

**Objective:**

A surge in parvovirus B19 infections has been reported in 2023–2024 across Europe and the USA, raising concerns about the associated perinatal risks. The aim of this study was to compare perinatal outcomes following maternal parvovirus B19 infection during the 2023–2024 period with those from a pre‐2023 cohort.

**Methods:**

This multicenter, retrospective cohort study compared perinatal outcomes in women with maternal parvovirus B19 infection according to whether infection occurred pre‐2023 (2012–2022) or between 2023 and 2024. Pregnant women with confirmed parvovirus B19 infection were eligible for inclusion. Cases were excluded if they had incomplete records, an ongoing pregnancy, coinfection with cytomegalovirus or Epstein–Barr virus, pre‐existing structural or genetic abnormality, immune fetal hydrops or a maternal serology result not indicative of parvovirus B19 infection. The primary outcome was perinatal mortality, which was defined as intrauterine fetal death ≥ 20 weeks' gestation or neonatal death ≤ 28 days after delivery. The secondary outcomes were persistent fetal anemia requiring more than one intrauterine transfusion (IUT) and a composite adverse perinatal outcome (CAPO), defined as the presence of one or more adverse outcomes, including perinatal mortality, pregnancy loss < 20 weeks, new‐onset structural anomaly and termination of pregnancy owing to parvovirus‐related morbidity. Differences between the two groups were assessed using standard statistical tests, and a generalized linear mixed model was used to identify predictors of perinatal mortality in the 2023–2024 cohort.

**Results:**

Following exclusions, 140 cases from pre‐2023 and 175 cases from 2023–2024 were analyzed. The rate of fetal hydrops at presentation was similar across the two groups (22.9% in pre‐2023 *vs* 23.4% in 2023–2024; *P* = 0.905). The rates of perinatal mortality (6.4% in pre‐2023 *vs* 8.0% in 2023–2024; *P* = 0.294) and CAPO (17.9% in pre‐2023 *vs* 21.7% in 2023–2024; *P* = 0.395) were not significantly different between groups, but the proportion of fetuses with persistent fetal anemia requiring a second IUT was significantly higher in the 2023–2024 cohort (46.0% *vs* 19.4%; *P* = 0.011). For the 2023–2024 cohort, fetal hydrops at presentation was an independent predictor of perinatal mortality (adjusted odds ratio, 10.91 (95% CI, 1.89–63.07); *P* = 0.007).

**Conclusion:**

In this multicenter collaboration, we report perinatal outcomes following maternal parvovirus B19 infection during the recent upsurge and compare them with those of a historical cohort. Although perinatal mortality and CAPO rates were similar between cohorts, cases in the recent surge (2023–2024) required more prenatal interventions, including the need for more than one IUT. Early identification and monitoring remain essential to mitigate adverse perinatal outcomes following maternal parvovirus B19 infection. © 2025 The Author(s). *Ultrasound in Obstetrics & Gynecology* published by John Wiley & Sons Ltd on behalf of International Society of Ultrasound in Obstetrics and Gynecology.

## INTRODUCTION

Parvovirus B19 is a small, single‐stranded DNA virus that primarily affects humans. It is best known as the cause of erythema infectiosum, commonly referred to as ‘fifth disease’ or ‘slapped‐cheek syndrome’, which is a mild illness predominantly affecting children. The virus is transmitted via respiratory droplets and is highly contagious. Parvovirus B19 infection is seasonal, with peak activity usually seen in spring and early summer, and cyclical peaks occurring every 3 to 4 years^1^. There has been low activity of this virus since 2018; however, there was an increase in reported cases of parvovirus B19 at the end of 2023 and the beginning of 2024^2^, ^3^. In most cases, parvovirus B19 infection is self‐limiting and asymptomatic, particularly in children and healthy adults^4^. However, the infection can have serious consequences for certain at‐risk groups, including pregnant women, immunocompromised individuals and those with a hemolytic disorder such as sickle cell disease^5^.

In pregnant women, parvovirus B19 can cross the placenta and infect the fetus, with vertical transmission occurring in approximately 33–51% of cases^6^. The virus targets erythroid progenitor cells in the fetal liver, leading to severe fetal anemia, hydrops fetalis and, in some cases, fetal demise. The risk of adverse outcome is highest when infection occurs between 9 and 20 weeks' gestation^7^. Fetal outcomes can be significantly improved with timely diagnosis and intervention, such as intrauterine transfusion (IUT), which has been shown to increase survival rates^8^.

In 2023–2024, there was an alarming surge in parvovirus B19 cases across Europe and the USA. Reports from the Centers for Disease Control and Prevention and various European health authorities have highlighted a significant increase in infection rates, particularly among children aged 5–9 years^9^, ^10^, ^11^. Similarly, a retrospective study analyzing 2.7 million electronic medical records in Israel from 2015–2023 found a marked increase in parvovirus B19 infections in 2023, with an adjusted incidence rate ratio (IRR) of 6.6 compared with previous years, and an IRR of 9.21 compared with the coronavirus disease 2019 (COVID‐19) period (2020–2021). The outbreak affected primarily high socioeconomic status groups and school‐aged children, with the most significant rise seen among pregnant women (IRR, 11.47) in 2023 compared with previous years, especially among those in the first trimester^12^. This surge was also accompanied by an increase in severe perinatal outcomes in pregnant women, including higher rates of fetal anemia and non‐immune hydrops fetalis requiring IUT^13^.

The current epidemic raises concerns about the possibility of a new, more virulent strain of parvovirus B19. While genotyping studies have not conclusively identified a new strain, the unusual epidemiological patterns and the severity of the outcomes observed suggest that changes in the behavior of the virus may be contributing to the increased rate of morbidity and mortality^13^, ^14^, ^15^. This hypothesis warrants further investigation, particularly considering the historical pattern of parvovirus B19 causing periodic epidemics.

Given the severity of the recent surge and the potential emergence of a more virulent strain, it is crucial to compare the perinatal outcomes of cases of parvovirus B19 infection in the current surge with those from historical cohorts.

Therefore, the primary aim of our study was to compare perinatal outcomes of cases of maternal parvovirus B19 infection in 2023–2024 with those from 2012–2022 (pre‐2023). The secondary objectives were to identify any significant changes in the clinical presentation or in the IUT management outcomes of parvovirus B19 infections in pregnancy.

## METHODS

This multicenter, retrospective cohort study included pregnant women with confirmed parvovirus B19 infection and compared such cases from 2023–2024 with a historical pre‐2023 cohort (2012–2022). Women were considered to have confirmed parvovirus B19 infection if serological evidence of maternal infection was present. In cases lacking reliable serological evidence, maternal infection was confirmed retrospectively based on sonographic findings indicative of fetal infection, which was subsequently validated through cordocentesis or amniocentesis. Cases were excluded from the analysis if they had an incomplete medical record, an ongoing pregnancy, co‐infection with cytomegalovirus or Epstein–Barr virus, pre‐existing structural or genetic abnormality, immune fetal hydrops or no evidence of maternal parvovirus B19 infection.

Data collection included demographic, clinical and outcome variables. Demographic characteristics included maternal age, body mass index and parity. Data on clinical presentation and disease‐related variables included gestational age (GA) at maternal infection, fetal hydrops at presentation and the need for IUT, with details recorded on the timing and frequency of transfusions.

The primary outcome was perinatal mortality, which was defined as intrauterine fetal death ≥ 20 weeks' gestation or neonatal death ≤ 28 days after delivery. The secondary outcomes were persistent fetal anemia requiring more than one IUT and a composite adverse perinatal outcome (CAPO), defined as the presence of one or more adverse outcomes, including perinatal mortality, pregnancy loss < 20 weeks, new‐onset structural anomaly, and termination of pregnancy (TOP) owing to parvovirus‐related morbidity. Additional fetal and neonatal outcomes, such as birth weight, GA at birth and admission to the neonatal intensive care unit, were recorded.

A typical management protocol for parvovirus B19 infection in pregnancy involves serological testing for immunoglobulin‐G and immunoglobulin‐M antibodies to confirm maternal infection once it is suspected. Following confirmation, serial ultrasound examinations (usually weekly, starting from 3–4 weeks after confirmed maternal infection) are recommended to monitor for signs of fetal anemia and hydrops, with Doppler assessment of the fetal middle cerebral artery peak systolic velocity to detect fetal anemia before the onset of hydrops. If fetal anemia is diagnosed, fetal blood sampling and, if indicated, IUT are conducted to manage severe cases^16^, ^17^. While this protocol formulates a standard approach, slight variations may exist across centers based on available resources and specific clinical guidelines.

No formal sample calculation was performed owing to the study's retrospective nature. A standardized Excel sheet (Microsoft, Redmond, WA, USA) was used to collect all required data variables. Anonymized data were obtained from electronic databases and recorded on the Excel sheet. The anonymized data were then transferred securely to the study coordinator (S.P.), who conducted the final analysis and stored the data on an encrypted hard drive. Preliminary analyses were performed using the Shapiro–Wilk test to examine variable distributions. Statistical analysis involved descriptive statistics to compare baseline characteristics and clinical outcomes between the pre‐2023 and the 2023–2024 groups. Median and interquartile range (IQR) were calculated for continuous variables, and *n* (%) was used for categorical variables. Differences between groups were assessed using the chi‐square or Fisher's exact test for categorical outcomes, and the Mann–Whitney *U*‐test was used for continuous outcomes. A generalized linear mixed model was used to assess predictors of perinatal mortality in the 2023–2024 group, incorporating both fixed and random effects to account for clustering within participating centers. The dependent variable was perinatal mortality, and the independent variables included maternal age, parity, need for IUT, GA at maternal infection and the presence of fetal hydrops at presentation. A binomial distribution with a logit link function was applied. Model parameters were estimated using the robust covariance matrix to account for within‐cluster correlation. All analyses were conducted using RStudio version 2024.12.0 + 467 (Posit PBC, Boston, MA, USA). Statistical significance was set at *P* < 0.05.

## RESULTS

**Figure 1:**
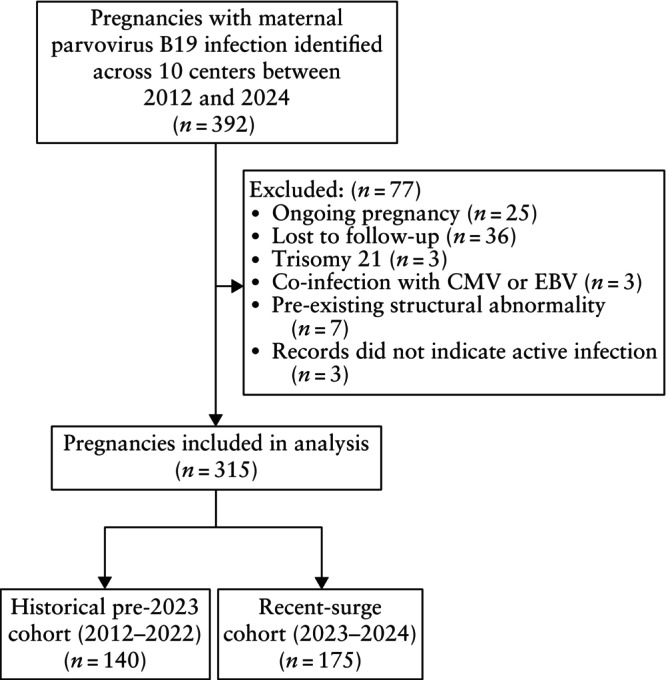
Flowchart showing inclusion of pregnancies with maternal parvovirus B19 infection in study. CMV, cytomegalovirus; EBV, Epstein–Barr virus.

A total of 392 cases of maternal parvovirus B19 infection were identified across 10 centers, of which 315 met the inclusion criteria (Figure 1). The participating centers are listed in Table S1. Exclusions were due to cases with trisomy 21 (*n* = 3), loss to follow‐up (*n* = 36), ongoing pregnancy (*n* = 25), coinfection with Epstein–Barr virus or cytomegalovirus (*n* = 3), pre‐existing fetal structural abnormality (*n* = 7) and records not indicative of active parvovirus B19 infection (*n* = 3). The total cohort in the analysis comprised 140 cases from the pre‐2023 period and 175 cases from the 2023–2024 period.

### Demographic and clinical characteristics

The demographic and clinical characteristics were similar across the two study groups (*P* > 0.05 for all) (Table 1). The median GA at maternal infection was 20.4 (IQR, 13.0–23.9) weeks for the pre‐2023 group and 20.8 (IQR, 15.6–24.0) weeks for the 2023–2024 group (*P* = 0.461). The timing of maternal infection by trimester distribution did not differ significantly between the two groups (*P* = 0.631). Fetal hydrops at presentation was observed in 22.9% of the pre‐2023 cases and 23.4% of the 2023–2024 cases (*P* = 0.905). There was no statistically significant difference in the need for IUT between the 2023–2024 and pre‐2023 groups (28.6% *vs* 25.7%; *P* = 0.572). However, persistent fetal anemia necessitating a second IUT was more common in the 2023–2024 infection group than in the pre‐2023 group (46.0% *vs* 19.4%; *P* = 0.011) (Table 2).

**Table 1:** Maternal and disease characteristics of pregnancies with parvovirus B19 infection, according to whether infection occurred pre‐2023 (2012–2020) or between 2023 and 2024

Characteristic	Pre‐2023 infection (*n* = 140)	2023–2024 infection (*n* = 175)	*P*
Maternal age (years)	33.0 (29.2–36.0)	33.0 (28.0–36.0)	0.721
Maternal BMI (kg/m^2^)	25.0 (22.3–28.7)	25.0 (22.2–28.6)	0.882
Nulliparous	47 (33.6)	43 (24.6)	0.079
Timing of maternal infection			0.631
First trimester	36 (25.7)	37 (21.1)	
Second trimester	87 (62.1)	116 (66.3)	
Third trimester	17 (12.1)	22 (12.6)	
GA at maternal infection (weeks)	20.4 (13.0–23.9)	20.8 (15.6–24.0)	0.461
Fetal hydrops at presentation	32 (22.9)	41 (23.4)	0.905
Need for IUT	36 (25.7)	50 (28.6)	0.572
GA at IUT (weeks)	22.0 (21.2–24.4)	23.3 (21.0–26.1)	0.402

Data are given as median (interquartile range) or *n* (%). BMI, body mass index; GA, gestational age; IUT, intrauterine transfusion.

### Perinatal outcomes

A comparison of the perinatal outcomes between the two groups is presented in Table 2. The rate of fetal loss following IUT did not differ significantly between pre‐2023 and 2023–2024 groups (19.4% *vs* 18.0%; *P* = 0.870). For those that resulted in live birth, the median GA at birth was 39.0 (IQR, 38.0–40.0) weeks in the pre‐2023 group and 38.0 (IQR, 35.9–38.7) weeks in the 2023–2024 group (*P* = 0.624). The median birth weight was also similar between the two groups (3000 (IQR, 2560–3270) g in the pre‐2023 group *vs* 2847 (IQR, 2534–3407) g in the 2023–2024 group; *P* = 0.243).There was no significant difference in the rate of perinatal mortality between the two study groups (6.4% in the pre‐2023 group *vs* 8.0% in the 2023–2024 group; *P* = 0.294). Furthermore, the live birth rate was similar between the two groups (89.3% in the pre‐2023 group *vs* 86.9% in the 2023–2024 group; *P* = 0.516), and there were no significant differences in the rate of pregnancy loss < 20 weeks, TOP or CAPO between the two groups (*P* > 0.05 for all).

**Table 2:** Comparison of perinatal outcomes of pregnancies with parvovirus B19 infection, according to whether infection occurred pre‐2023 (2012–2022) or between 2023 and 2024

Outcome	Pre‐2023 infection (*n* = 140)	2023–2024 infection (*n* = 175)	*P*
Fetal loss after IUT	7/36 (19.4)	9/50 (18.0)	0.870
GA at live birth (weeks)	39.0 (38.0–40.0)	38.0 (35.9–38.7)	0.624
Birth weight (g)	3000 (2560–3270)	2847 (2534–3407)	0.243
Persistent fetal anemia needing second IUT	7/36 (19.4)	23/50 (46.0)	0.011
Persistent fetal anemia needing three or more IUTs	0/36 (0.0)	11/50 (22.0)	0.003
Time for resolution of fetal anemia (days)	17.0 (7.0–29.5)	28.0 (14.0–56.0)	0.038
Live birth	125 (89.3)	152 (86.9)	0.516
Pregnancy loss < 20 weeks	2 (1.4)	4 (2.3)	0.260
TOP*	4 (2.9)	5 (2.9)	1.000
IUFD ≥ 20 weeks	8 (5.7)	11 (6.3)	0.824
NND ≤ 28 days after delivery	1 (0.7)	3 (1.7)	0.831
Perinatal mortality†	9 (6.4)	14 (8.0)	0.294
CAPO‡	25 (17.9)	38 (21.7)	0.395

Data are given as *n*/*N* (%), median (interquartile range) or *n* (%).

* Termination of pregnancy (TOP) owing to parvovirus‐related morbidity.

† Perinatal mortality was defined as intrauterine fetal death (IUFD) ≥ 20 weeks' gestation or neonatal death (NND) ≤ 28 days after delivery.

‡ Composite adverse pregnancy outcome (CAPO) was defined as the presence of one or more adverse events, including perinatal mortality, pregnancy loss < 20 weeks, new‐onset structural anomaly and TOP owing to parvovirus‐related morbidities. GA, gestational age; IUT, intrauterine transfusion.

### Factors associated with perinatal mortality

A generalized linear mixed model was performed to identify predictors of perinatal mortality in the 2023–2024 group (Table 3). Variance of the random effect was estimated as zero, indicating no significant center‐level variation. The presence of fetal hydrops at the time of presentation was found to be a significant predictor of perinatal mortality (adjusted odds ratio (aOR), 10.91 (95% CI, 1.89–63.07); *P* = 0.007), although this analysis was underpowered. Other variables, including maternal age (aOR, 0.99 (95% CI, 0.88–1.12); *P* = 0.919), parity (aOR, 0.80 (95% CI, 0.20–3.18); *P* = 0.753), need for IUT (aOR, 1.13 (95% CI, 0.20–6.37); *P* = 0.887) and GA at maternal infection (aOR, 0.96 (95% CI, 0.86–1.07); *P* = 0.451) were not significantly associated with perinatal mortality.

**Table 3:** Univariate and multivariate generalized linear mixed model (GLMM) analysis of predictors of perinatal mortality following maternal parvovirus B19 infection in recent surge in 2023–2024

	Univariate GLMM			Multivariate GLMM		
Predictor	Estimate	OR (95% CI)	*P*	Estimate	aOR (95% CI)*	*P*
Maternal age (years)	–0.065	0.937 (0.844–1.040)	0.227	–0.006	0.993 (0.880–1.122)	0.919
Parity	−0.656	0.519 (0.163–1.646)	0.266	–0.220	0.802 (0.202–3.178)	0.753
GA at maternal infection (weeks)	−0.009	0.991 (0.916–1.072)	0.816	–0.043	0.957 (0.855–1.071)	0.451
Fetal hydrops	2.448	11.570 (3.380–39.620)	< 0.001	2.389	10.905 (1.885–63.068)	0.007
Need for IUT	1.686	5.400 (1.700–17.100)	0.004	0.124	1.132 (0.201–6.366)	0.887

* Adjusted for maternal age, parity, gestational age (GA) at maternal infection, fetal hydrops and need for intrauterine transfusion (IUT). GLMM was performed with ‘participating center’ as a random effect, however, variance was estimated as zero, indicating no significant center‐level variation. aOR, adjusted odds ratio; OR, odds ratio.

## DISCUSSION

### Main findings

The present study found that while demographic and clinical characteristics, perinatal mortality and CAPO were largely similar between the pre‐2023 and 2023–2024 groups, cases of maternal parvovirus B19 infection in 2023–2024 were more likely to need more than one IUT. Perinatal outcomes, including fetal loss after IUT, GA at birth and live birth rate, remained comparable between the two groups. Furthermore, in the 2023–2024 cohort, the presence of fetal hydrops at presentation was identified as a significant independent predictor of perinatal mortality.

### Interpretation in the context of the existing literature

To date, limited data are available on maternal parvovirus B19 infection in pregnancy during the recent surge in cases, particularly regarding its impact on perinatal outcomes. The recent surge has brought significant attention to the heightened risk of adverse perinatal outcomes associated with maternal parvovirus B19 infection, especially in fetuses with severe anemia and hydrops fetalis^18^, ^19^. Nordholm *et al*.^20^ reported on an epidemic of parvovirus B19 and the severity of the associated disease in a cohort in Denmark, in which 130/648 (20.1%) laboratory‐confirmed cases occurred in pregnant individuals. Among these cases, 12.3% (16/130) experienced a severe adverse outcome, including fetal anemia, hydrops fetalis and miscarriage. Our recent‐surge cohort (2023–2024) observed a CAPO rate of 21.7% and a perinatal mortality rate of 8.0%.

Our study found fetal hydrops at presentation to be the only significant predictor of perinatal mortality in the recent cohort (aOR, 10.91 (95% CI, 1.89–63.07); *P* = 0.007). The meta‐analysis of Bascietto *et al*.^7^, which examined 127 studies involving over 1700 cases of parvovirus B19 infection, reported a markedly elevated risk of adverse outcome in hydropic fetuses compared with non‐hydropic cases; the mortality rate in hydropic fetuses was 55% compared with 11% in non‐hydropic cases. Furthermore, in cases for which an IUT was required, the survival rate improved significantly but remained suboptimal without early detection and intervention. The increased need for more than one IUT and the longer time period for resolution of anemia may be markers of disease severity in our cohort. However, the overall rate of perinatal mortality and CAPO did not significantly differ between the pre‐2023 and the 2023–2024 groups. This observation may be attributed to intervention bias, as these pregnancies were actively identified, closely monitored and appropriately treated. In our study, there was no significant difference in the GA at IUT between the two groups (*P* = 0.402). In contrast, Russcher *et al*.^13^ reported a significantly higher GA at first IUT in cases with adverse outcomes (mean, 22.8 weeks) compared to those without (mean, 20.8 weeks) (*P* = 0.010).

In a Northwestern European cohort, during the 2023–2024 parvovirus B19 upsurge, 36% of fetuses receiving IUTs experienced adverse outcomes, including perinatal death (22%), TOP owing to severe anomaly (7%) and ongoing pregnancy with persistent hydrops or cerebral anomaly (7%)^13^. Tassis *et al*.^21^ reported similar trends in which the 2024 parvovirus B19 outbreak in Northern Italy demonstrated a dramatic rise in infections, with 59 cases identified in the first 7 months of 2024 compared with fewer than seven cases per year in the preceding period (2015–2023). Despite this increase, maternal characteristics and fetal outcomes, including fetal anemia and adverse events, did not differ significantly between the outbreak period and earlier years. In our study, we observed similar trends, with a similar rate of CAPO in the 2023–2024 cohort (21.7%) compared with the pre‐2023 group (17.9%).

### Clinical and research implications

The post‐COVID‐19‐pandemic period may have altered the parvovirus B19 infection dynamics, influenced by shifts in social behavior and a potential loss of population‐level immunity owing to prolonged isolation^22^, ^23^. These factors may have contributed to the recent surge in maternal parvovirus B19 infections observed across multiple regions. Unlike for other infections, there is currently no effective primary prevention for parvovirus B19; thus, identifying infected mothers through seroconversion remains essential^17^. Early detection enables timely surveillance of fetuses at risk, which is crucial as advanced disease manifestations, such as fetal hydrops, are associated with severe adverse outcomes, including acquired brain lesions, increased risk of perinatal mortality and long‐term neurodevelopmental impairment.

From a research perspective, concerted efforts are needed to advance our understanding of the behavior of parvovirus B19, including its temporal patterns and the potential changes in virulence or genotype. Investigating whether emerging variants alter disease severity or prolong recovery will be crucial for future outbreak preparedness. Collaborative multicenter research initiatives could provide valuable data on infection rates, clinical course and perinatal outcomes, facilitating more robust guidelines for managing these cases. Developing vaccines to provide immunity against parvovirus B19 for women of childbearing age would be a significant advance, potentially preventing fetal complications associated with congenital infection^24^.

### Strengths and limitations

This study has several strengths. It represents a large, multicenter collaboration that provides timely insights into the outcomes of the recent parvovirus B19 infection surge, drawing on data from maternal–fetal medicine centers across regions experiencing increased infection rates. By including data from specialist tertiary centers, this study offers a robust overview of how high‐risk cases have been managed during this period. Moreover, the data reflect real‐world clinical practice in regions in which the surge is ongoing, which enhances the study's relevance and applicability to current clinical contexts.

However, several limitations must be considered. The sample size, while informative, was still limited and may have affected the generalizability of our findings to broader populations. Furthermore, the retrospective nature of the study has inherent bias, and the data were collected over a considerable period of time, during which monitoring and management protocols for parvovirus B19 infection may have evolved. Advances in IUT techniques, increased operator expertise and improved ultrasound technology may have influenced outcomes over time, potentially confounding the comparisons across cases. Additionally, heterogeneity in protocols for monitoring and managing infection across centers may have introduced outcome variability. Reliable data on procedural details and disease characteristics, such as the prevalence of concomitant thrombocytopenia and persistent fetal hydrops at birth, were not available for analysis. Thrombocytopenia is a recognized complication of fetal parvovirus B19 infection, with reported incidences ranging from 29–64%, and severe cases may require intrauterine platelet transfusion^25^. However, in our cohort, reliable data on platelet transfusion and thrombocytopenia were available for only 74 out of 315 cases, preventing robust analysis of its impact on perinatal outcomes. These factors collectively restrict the applicability and generalizability of our findings and should be considered when interpreting the results.

Finally, long‐term outcomes were not included in this analysis, as the primary objective was to address the impact of the recent surge in parvovirus B19 infections. Despite these limitations, our cohort provides valuable data and insights into the effects and management of parvovirus B19 in pregnancy during the recent surge.

### Conclusion

While the overall rate of adverse perinatal outcomes remained stable in the 2023–2024 cohort, cases in this recent surge of parvovirus B19 infections appear more severe and were more likely to need more than one IUT compared with the pre‐2023 cohort. Fetal hydrops at presentation was a key predictor of perinatal mortality, highlighting the need for early identification and close monitoring to enable timely intervention. Standardizing clinical protocols and advancing research, especially in vaccine development, are crucial for improving pregnancy outcomes in cases of parvovirus B19 infection.

### Collaborators

**C. Ozan Ulusoy**, Ministry of Health, Etlik City Hospital, Perinatology Department, Ankara, Turkey

**D. Mohammed**, Fetal Medicine Unit, St George's University Hospital, London, UK

**M. Arora**, Fetal Medicine Unit, St George's University Hospital, London, UK

**A. Hegde**, Fetal Medicine Unit, St George's University Hospital, London, UK

**E. Klapholz**, Department of Obstetrics and Gynecology, Sheba Medical Center, Faculty of Medical and Health Sciences, Tel Aviv University, Tel Aviv, Israel

**C. Berkovitz**, Department of Obstetrics and Gynecology, Sheba Medical Center, Faculty of Medical and Health Sciences, Tel Aviv University, Tel Aviv, Israel

**K. Kosińska‐Kaczyńska**, Department of Obstetrics, Perinatology and Neonatology, Centre of Postgraduate Medical Education, Warsaw, Poland

**I. Rzucidło‐Szymańska**, Department of Obstetrics, Perinatology and Neonatology, Centre of Postgraduate Medical Education, Warsaw, Poland

**N. Szymecka‐Samaha**, Department of Obstetrics, Perinatology and Neonatology, Centre of Postgraduate Medical Education, Warsaw, Poland

**S. Ceffa**, Prenatal Medicine Unit, Obstetrics and Gynaecology Unit, Department of Medical and Surgical Sciences for Mother, Child and Adult, University of Modena and Reggio Emilia, Modena, Italy

## Supporting information


**Table S1** List of participating centers

## Data Availability

The data that support the findings of this study are available from the corresponding author upon reasonable request.
