# Supplementary material for: Is parvovirus B19 infection upsurge in 2023–2024 associated with adverse pregnancy outcome?
Source: Ultrasound Obstet Gynecol. 2025 Jul 30;66(3):307–13. doi: 10.1002/uog.29301 (PMC12401498; doi:10.1002/uog.29301)
Supplement: Supplementary file 1 — Table S1 List of participating centers [file UOG-66-307-s001.docx]

**Supplementary Table 1**  List of participating Centers

| **Participating Center** | **City and Country** |
| --- | --- |
| St. George’s University Hospital | London, UK |
| Sheba Medical Center, Tel Hashomer Hospital | Tel Aviv, Israel |
| Centre of Postgraduate Medical Education | Warsaw, Poland |
| Centre Pluridisciplinaire de Diagnostic Prénatal Centre Hospitalier Lyon Sud, Université Claude Bernard Lyon 1 | Villeurbanne, France |
| Institute of Mother and Child, ul. Kasprzaka 17a, 01-211 Warsaw | Warsaw, Poland |
| University of Modena and Reggio Emilia | Modena, Italy |
| University of Chieti | Chieti, Italy |
| IRCCS Istituto Giannina Gaslini | Genova, Italy |
| Medical University of Warsaw | Warsaw, Poland |
| Hospital Universitario y Politécnico La Fe | Valencia, Spain |
